# Hurdle technology using enzymes and essential oil to remove biofilm and increase the effectiveness of this process with the microencapsulation method

**DOI:** 10.1002/fsn3.4377

**Published:** 2024-08-28

**Authors:** Ayda Ghahari, Kianoush Khosravi‐Darani

**Affiliations:** ^1^ Bioprocess Engineering Department Institute of Industrial and Environmental Biotechnology, National Institute of Genetic Engineering and Biotechnology Tehran Iran; ^2^ Research Department of Food Technology, National Nutrition and Food Technology Research Institute, Faculty of Nutrition Sciences and Food Technology Shahid Beheshti University of Medical Sciences Tehran Iran

**Keywords:** biofilm, enzyme, essential oil, hurdle technology, microencapsulation

## Abstract

The formation of biofilm in different places and the failure to effectively remove it by the usual disinfection methods is due to its structure and the rich genetic resource available in it to deal with disinfectants. These impenetrable structures and diverse microbial genetics have caused biofilm pollution in different industries like the food industry, the medicine industry, the hospitals and the water distribution system, resulting in pathogenicity and reduction of industrial quality. An efficient way to deal with the resistant population of biofilm‐forming microbes is the use of hurdle technology including enzymes and essential oils. Enzymes reduce the resistance of the biofilm structure due to degradation of its extracellular polymer matrix (EPS) by their abilities to break down the organic molecules, and then the essential oils weaken the cells by penetrating the lipid membrane of the cell and destroying its integrity; as a result, the biofilm will be destroyed. The advantage of this hurdle technology is the environmental friendly of both methods, which reduces concerns about the use of chemical disinfection methods, but on the other hand, due to the sensitivity of enzymes as biological agents also the expensiveness of this technique and the considerations of working with essential oils as volatile and unstable liquids should abandon the routine methods of applying this disinfectant to biofilm and go for the microencapsulation method, which as a protective system increases the effectiveness of enzymes and essential oils as antibiofilm agents.

## Graphical abstract

Combine effect of enzymes, essential oil via microencapsulation technique.

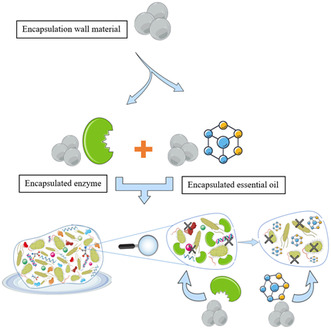

## 1 INTRODUCTION

One of the most serious well‐being dangers is the formation of biofilm in several places, including treatment places, industrial equipment, the water distribution system and places related to nourishment, which causes sickness, mechanical blockage, energy loss and a decrease in material quality (Abdallah et al., 2014; Carrascosa et al., 2021). Within the field of nourishment diseases caused by biofilms, Centers for Disease Control and Prevention (CDC, 2019), presented 841 sorts of contaminations in 2017 (CDC, 2019) that many of them are caused by improper storage and transportation of food (Ghahary & Abiri, 2021). Moreover, healing center diseases are the foremost common side impact within the field of health; many patients get various infections and sometimes die when they go to medical centers and hospitals. The reason for many of these infections is biofilms that cannot be removed by conventional methods (Khatoon et al., 2018).

Microscopic organisms (such as bacteria, fungi and algae) tend to connect to non‐living surfaces and shape biofilms; within the differing world of microscopic organisms, 40%–80% of them can form biofilms (Flemming & Wuertz, 2019). Biofilms are a population of microbes that attach to a surface and cooperate to create a three‐dimensional network that secures the cells inside the biofilm structure, and the high genetic diversity of these cells together causes their strong resistance against anti‐microbials, biocides and UV radiation (Bridier & Briandet, 2022; Xu et al., 2017). In the biofilm structure, the rate of production of secondary metabolites and their discharge, as well as the rate of genetic exchanges, is very high (Rodrigues & Černáková, 2020). The increase in the production rate of secondary metabolites occurs under the influence of a process called Quorum Sensing (QS), in which biofilm‐forming cells send signals in response to external and internal stimuli. A variety of Quorum Sensing signals are divided into these groups: synthesis of secondary metabolites by the biofilm microbial population and the conduction of these metabolites, acquisition of the microbiome nutrients requirements, cellular pathways regulation, defense processes control, genetic transfer regulation and conduction, and the modulation of biofilm pathogenicity (Al‐Azzawi et al., 2024). These signals are capable to transfer information through the formation of particles called autoinducers. These autoinducers cause continuous changes in the behavior and structure of the biofilm under the influence of conditions, so that the microbial population forming this biofilm can protect itself against internal and external stresses such as competing microbes. In gram‐negative microbes, autoinducers are acylated homoserines, and in gram‐positives, they are oligopeptides that inhibit or activate the expression of certain genes under the influence of specific concentrations and intensities of their stimuli (Padder et al., 2018; Zhao et al., 2020).

Until nowadays, numerous strategies have been utilized to control biofilms, which drop into two categories, chemical and physical. Chemical strategies such as surfactants, detergents, biocides and physical strategies such as sonication, melting and freezing, but none of these strategies have been beneficial sufficient so distant (Roy et al., 2018). Among all the methods used, disinfection is one of the best biofilm control strategies, but since one of the most important reasons for the development of antimicrobial resistance in the microbial population is the use of these disinfectants, it is better to go for other methods to prevent side effects of biofilm removal (Bayoumi et al., 2012). For the reasons mentioned, the need to move toward new technologies is obvious. Hurdle technology, meaning the use of two or more distinct strategies simultaneously to enhance effectiveness, can be very effective in removing biofilms (Khan et al., 2017). For example, Table 1 shows utilize of hurdle technology to evacuate biofilms from nourishment sources.

**TABLE 1:** Using hurdle technology to remove food biofilms.

Methods	Food type	Removed microorganism	Microorganism reduction after treatment
Pressure + heat treatment (Nabi et al., 2021)	Juice	*Salmonella*	7 CFU/mL
Electron beam irradiation + cold treatment (Tolentino et al., 2021)	Fresh beef	*The entire microbial population*	2 CFU/g
Cellulase + cetyltrimethylammonium bromide (Wang et al., 2016)	Meat	*Salmonella*	6.22 log CFU/cm^2^ (completely remove biofilm)
Clove oil + cold nitrogen plasma (Cui, Zhou, et al., 2016)	Milk	*Escherichia coli O157:H7*	5.48 log CFU/cm^2^

**FIGURE 1:**
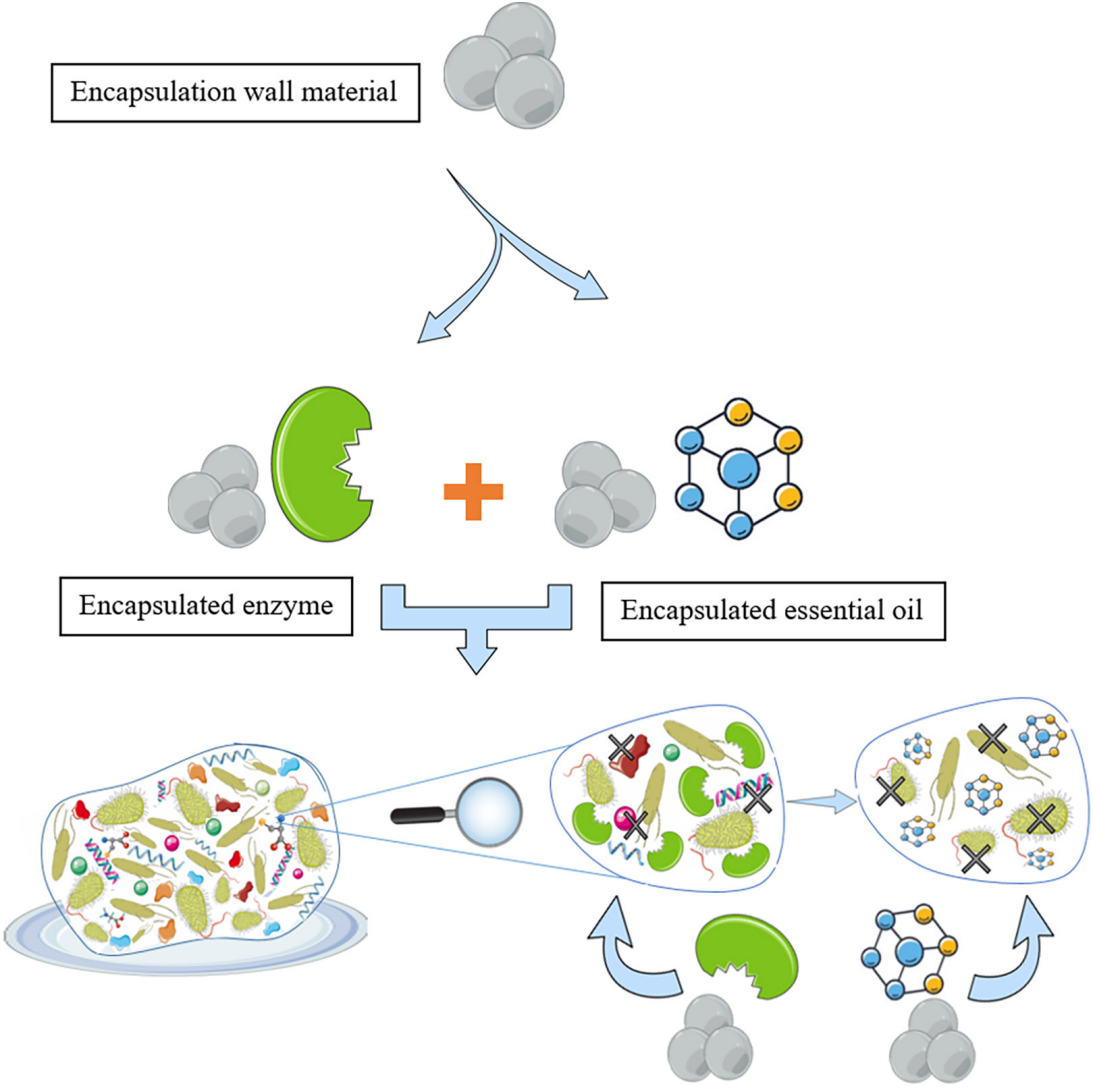
Hurdle technology using microencapsulated enzyme and essential oil to remove biofilm. https://bioicons.com/.

A new but very effective hurdle technology to destroy biofilms is a combination of enzymes and essential oils that are treated with biofilm, respectively, and works strategically to weaken and then destroy the biofilm. In this technology, enzymes destroy the high resistance of the biofilm matrix structure by breaking down the biomolecules in this matrix into their components. This causes the loss of matrix integrity and thus the loss of the protective framework of the biofilm. Of course, enzymes moreover make cells more prepared for lysis. Now, essential oil as an organic antimicrobial can penetrate the weak structure of the biofilm and cause one or more changes: (1) penetrating the cell membrane and changing the integrity of this membrane, (2) activating apoptosis signaling pathways, (3) creating changes in the expression of genes and (4) changing the ratio of lipids in the membrane or cell wall, which ultimately, each of these cases leads to a change in the balance of the cell and death (Gao et al., 2024; Mohamed et al., 2018). Essential oils are fragrant and oily fluids that are isolated from plant materials that their antibacterial capacities have been known for a long time and their efficacy against pathogenic microbes and fungi has been demonstrated (Oulkheir et al., 2017), but there are challenges to utilizing these oils, for example, low solvency in water, low stability, being volatile, and sensitivity to light and heat (Wang, Zhao, et al., 2023). To solve these challenges, a technique called microencapsulation can be used which largely eliminates the limitations of using essential oils and enzymes and causes the effective and strategic performance of these two agents; in fact, sequential use of encapsulated enzymes and then the encapsulated essential oils makes each of them able to have the greatest effect on their target compounds (Figure 1) (Cui, Li, et al., 2016; Sousa et al., 2022). These microcapsules are basically liposomal carriers, solid lipid nanoparticles and polymeric particles such as gelatin nanoparticles (El Asbahani et al., 2015), Arabic gum (Lv et al., 2014) and alginate microparticles (Hosseini et al., 2013).

## 2 BIOFILMS: COMPOSITION, ARCHITECTURE AND FORMATION

Biofilm is a three‐dimensional microbial populace that lives on a surface and interior of a hydrated matrix called extracellular polymer matrix (EPS); on the one hand, this microbial population can be homogeneous and contain only one strain of that microbe type, on the other hand, it is possible to be heterogeneous and contain more than one strain of one type of microbe, even one or more strains of different types of microbes (such as bacteria and fungi simultaneously). Due to this diversity, microbial populace is totally non‐uniform in terms of behavior and genetics that can cause the advancement of new strains through genetic exchanges (Abdallah et al., 2014; Rodrigues & Černáková, 2020). EPS is much hydrated, and its major portion is made up of proteins (particularly amyloid fibers), polysaccharides, RNA and DNA, lipids, glycolipids, extracellular enzymes, surfactants and ions; these compounds generally depend on the strain and species of bacteria and the environmental conditions (Flemming & Wuertz, 2019). This matrix causes the structural integrity of the biofilm and secures it against environmental stresses such as osmotic shock, dryness, pH changes, rays such as UV rays and temperature changes (Flemming et al., 2016; Karygianni et al., 2020).

**FIGURE 2:**
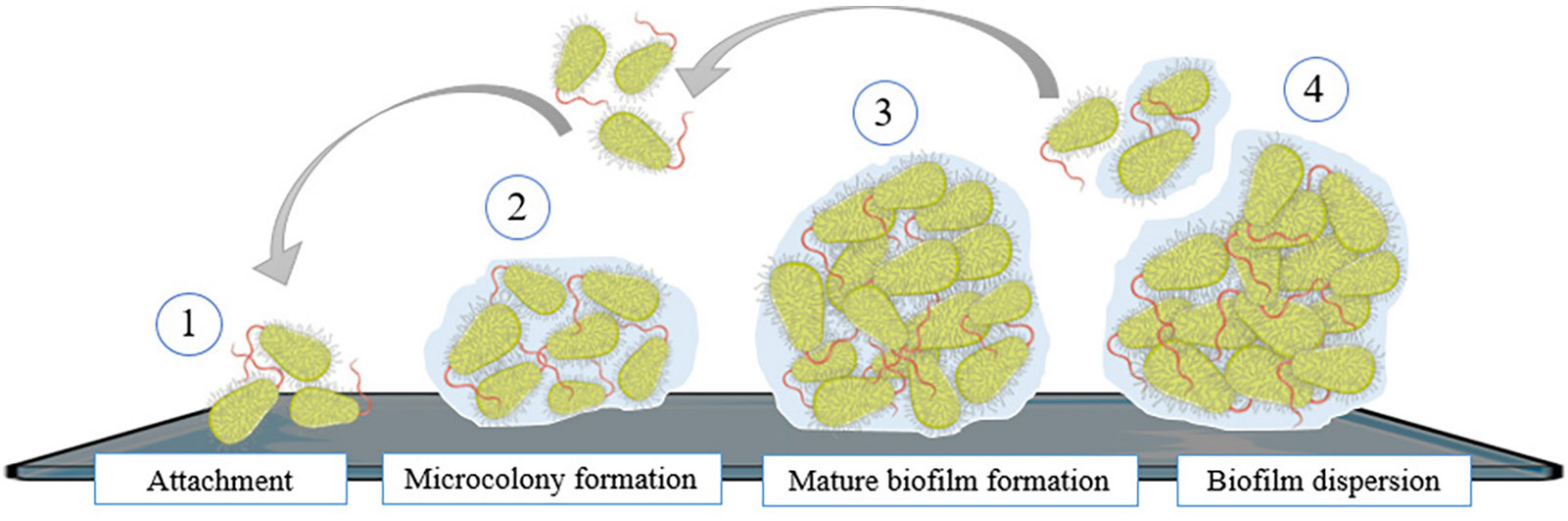
Stages of biofilm formation. https://bioicons.com/.

Generally, biofilm formation takes place in four main stages (Figure 2); but it is worth mentioning that these stages could vary in detail, according to a certain microbe type or strain. These four stages consist of the following:

First stage: attachment; microbes are attached to the surface with the help of their appendages, and these connections are reversible and non‐specific, such as Van der Waals and hydrophobic connections.

Second stage: micro‐colony formation; the connection becomes stronger, and microbes begin to multiply, divide, and connect to each other and then begin to build the necessary precursors to form the matrix.

Third stage: formation of mature biofilm; the number of microbes increases through multiplication and division, and the matrix is formed, so the three‐dimensional structure of the biofilm is created. Also, at this stage, chrome sensing (QS) and gene transfer peak.

Fourth stage: due to various reasons such as mechanical disturbances, enzymatic decomposition of the matrix or the production of surfactant by cells, single cells separate from the biofilm, attach to the new surfaces and create the central nucleus of the new biofilm. These separated cells have changed in terms of resistance or pathogenicity compared to their free form (Goel et al., 2021; Khelissa et al., 2017; McDougald et al., 2012).

So, concurring with these explanations, biofilm removal is considered essential. Researches have appeared that disinfectants have a more noteworthy effect on the free form of microbes than biofilms. For case, chlorine‐containing disinfectants have a great effect on the free form, but they have almost no effect on biofilms, and decades of using such disinfectants only have increased the resistance of biofilm microbial populations (Wang, Wang, et al., 2023). Therefore, must go for organic methods like enzymes and essential oils; in this way, worldwide concerns and environmental damage caused by physical and chemical treatments can be decreased.

## 3 ENZYMES: FIRST REACTANT IN HURDLE TECHNOLOGY

Enzymes, by digesting extracellular polymeric matrix materials, convert them into their constituent units, which are easily consumed by biofilm cells and thus destroy the protective skeleton of this structural population (Simoes et al., 2020). By using enzyme as a biodegradable pretreatment to remove EPS, biofilm can be destroyed more effectively with lower concentrations of biocide (Meireles et al., 2016). Enzymes can play a role in biofilm degradation in several ways: direct attack on the matrix and its degradation, increasing cell lysis by weakening the cell wall, blocking the quorum sensing system and catalytic role for antimicrobial production reactions by biofilm cells (Augustin et al., 2004; Thallinger et al., 2013). Therefore, different enzymes are necessary to achieve each of the mentioned effectivenesses; four general categories of enzymes are used for this purpose, including proteolytic enzymes, anti‐Quorum sensing enzymes, carbohydrases and oxidizing enzymes in three classes: lyases, hydrolases and oxidoreductases (Thallinger et al., 2013).

In one study, a cocktail of carbohydrases isolated from *Aspergillus niger* has treated with biofilms formed by *Salmonella enterica serovar Typhi*, *Escherichia coli* and *Staphylococcus aureus* that caused 90.23 ± 4.0%, 82.64 ± 5.0% and 76.32 ± 5.0% reduction of the biofilms formed by these organisms, respectively. In addition to showing the effect of enzymes as an effective biocide on biofilm removal, this study can prove that the use of a mixture of enzymes (here, carbohydrases) can be more effective because of heterogeneous nature of the organic molecules in biofilm matrix (Kaur et al., 2020). Also, there are other examples of using enzyme treatment to remove biofilms in food industry, in Table 2.

**TABLE 2:** Using enzyme treatment to remove biofilms in food industry.

Applied enzyme	Enzyme class	Food or material type	Target biofilm	References
Flavourzyme	Peptidase	Food‐contact surfaces (ultra‐high–molecular‐weight polyethylene (UHMWPE) and rubber surfaces)	*Typhimurium, Escherichia coli, Pseudomonas aeruginosa*	Nahar et al. (2021)
Mannanase + Savinase + α‐amylase	Glycosyl hydrolase, Proteolytic, Glycoside hydrolase (respectively)	Pork contact surfaces	*Listeria monocytogenes*	Ripolles‐Avila et al. (2020)
DNase I + Proteinase K	Nuclease, serine protease (respectively)	Food‐grade stainless steel	*Listeria monocytogenes*	Nguyen and Burrows (2014)
DNase I, proteinase K, dispase II, cellulase, α‐amylase	Nuclease, serine protease, protease, glycoside hydrolase (respectively)	Food‐contact surfaces (polystyrene)	*Bacillus cereus*	Lim et al. (2021)
Lysostaphin	*Glycylglycine endopeptidase*	Diary	*Staphylococcus aureus*	Ceotto‐Vigoder et al. (2016)
Papain	Cysteine proteinase	Meat	*Acinetobacter, S. aureus*	Manohar et al. (2015)

Research on the use of enzymes to destroy the biofilm population has progressed so much that recently researchers have designed synthetic enzymes with higher stability for the purpose of biofilm removal and have named them the new generation of antibiotics (Chen et al., 2018). But enzymes, such as green biocides, have a higher price than chemical biocides, and this makes them still not used routinely (Satpathy et al., 2016).

## 4 ESSENTIAL OILS: FINAL REACTANT IN HURDLE TECHNOLOGY

Essential oils are volatile fluids (due to the presence of compounds such as phenol, alcohol, terpene and ester) and aromatics extracted from plants, whose components depend on its plant sources (Reyes‐Jurado et al., 2015). The hydrophobicity of these compounds is their most critical feature, which causes this oil to enter into the cellular lipid membrane and destroy its integrity, causing ions and molecules interior the cell to spread and leak into the outside space. As a result, the cell dies due to the loss of intracellular balance. Among these hydrophobic substances found in oil, phenolic compounds have special antimicrobial and antioxidant action. This hydrophobicity is so effective that Gram‐positive bacteria are more destroyed by essential oils due to the absence of a lipopolysaccharide membrane, and Gram‐negative bacteria are less accessible to these essential oils due to the presence of this membrane (Rao et al., 2019). In addition to the ability to remove formed biofilms, essential oils have also shown the ability to prevent the formation of biofilms. For example, in a research using eugenol and Cinnamomum zeylanicum essential oils, they were able to effectively prevent the formation of *E. coli* and *S. aureus* biofilms (Millezi et al., 2019). There are other examples of using essential oils as antibiofilm agents in food industry, shown in Table 3. One of the most prominent advantages of using essential oils as biocides is that both their liquid form and their vapors contain a mix of antimicrobial compounds, which makes it possible to use these vapors in places containing biofilm where access is limited (Antih et al., 2021).

**TABLE 3:** Using essential oil treatment to remove biofilms in food industry.

Essential oil	Essential oil resource	Food or material type	Target biofilm	References
Clove + oregano essential oils	*Syzygium aromaticum, Origanum vulgare*	Beef	*Salmonella derby*	Liu et al. (2022)
Carvacrol	Lamiaceae species	Polypropylene, stainless steel	*Salmonella typhimurium*	Trevisan et al. (2018)
Cardamom essential oil	*Elettaria cardamomum*	Different food container materials (plastic, steel and glass)	*Staphylococcus aureus*	Cui et al. (2020)
Mandarin essential oil	*Citrus reticulata*	Pork	*Staphylococcus aureus*, *Escherichia coli*	Song et al. (2021)
Garlic + thyme essential oils	Garlic*, Thymus vulgaris L. and Thymus zygis L*. (respectively)	Beef carcasses and the surfaces of slaughterhouse	*Salmonella typhimurium*	Morshdy et al. (2022)

## 5 NECESSITY OF USING HURDLE TECHNOLOGY

Subsequently, it can be demonstrated that both enzymes and essential oils independently can be used to remove and control biofilms, but there are logical reasons for using hurdle technology and combining these two strategies, which makes us move toward it. One of the reasons is that the optimal conditions for enzyme activity are not available in industrial environments, so they must be combined with an auxiliary biocide to get a higher efficacy method. And on the other hand, a large amount of essential oil treated with biofilm is non‐specifically attached to the components of the matrix, and its effectiveness is greatly reduced. It is also desirable that the biocide can perform optimally in the shortest time, the lowest concentration, the lowest number of times of use, and the lowest amount of waste production and without damage to the equipment. Achieving these goals is not possible without combining different methods, and if these methods are biodegradable and safe and consume little energy, they will be very welcome (Kim et al., 2019; Mechmechani et al., 2022).

For understanding the effect of using two or more methods for removing progressive microbial population of biofilms should attend to an example that shows this difference. A research in 2018 showed that the simultaneous use of hydrogen peroxide and quaternary ammonium disinfectants, or using octanoic acid and peracetic acid instead of quaternary ammonium disinfectants in this combination, can lead to the destruction of the entire bacterial population of *Listeria monocytogenes* in its homogenous biofilm, while the use of any of the mentioned disinfectants alone cannot create a significant effect in destroying this biofilm (Dhowlaghar et al., 2018) (also there are other examples in Table 1).

## 6 MICROENCAPSULATION, CONTROL CARRIER IN HURDLE TECHNOLOGY

**FIGURE 3:**
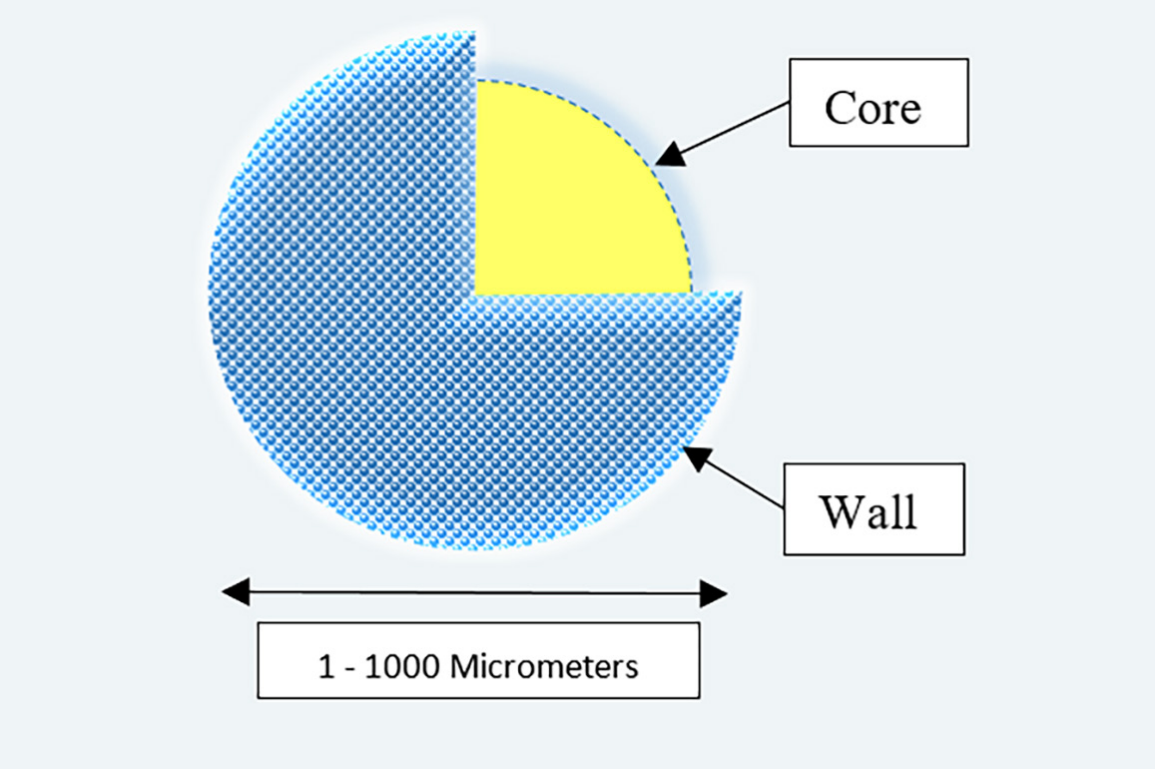
Microcapsules structure.

The simultaneous use of hurdle technology and microencapsulation means to enclose materials in particles with a size range of 1–1000 μm (Fu & Hu, 2017). Choosing the wall of this capsule may be a very sensitive and critical choice that must have certain characteristics such as non‐reaction with the core (substances within the capsule), protecting the core and the proper control of secretion and release in all environmental conditions (Figure 3) (Dhakal & He, 2020; Nazzaro et al., 2012). These used materials incorporate natural polymers and synthetic polymers; natural polymers include proteins such as collagen, carbohydrates such as gelatin and modified carbohydrates such as polydextran. Also, synthetic polymers are separated into two categories: non‐biodegradable synthetic polymers such as acrolein and biodegradable such as glycolides (Kowalska et al., 2022).

## 7 RELEASE PRINCIPLES

Several variables affect the release rate of the core from the capsule, the most important of which are the viscosity of the capsule wall material, the ratio of the core to the wall material, the interactions between the core and the wall, and the size of the particles forming the wall. If all these variables are suitable for the purpose of microencapsulation, the release will take place at the right time and place and at the right speed. Release process can occur via one or more stimulus including degradation of wall material by enzymes like lipases, dissolution of wall materials, diffusion of wall in aqueous or organic environment based on wall material, melting or expanding of wall in high temperature, pH changes that can alter the wall material solubility, and applying pressure to break down the wall material and release the core (Choudhury et al., 2021; Frascareli et al., 2012; Lengyel et al., 2019; Silva et al., 2014).

For example, in the research that was done for the encapsulation of juniper berry essential oil, they used different materials as walls in making capsules including gum arabic, maltodextrin, low viscosity sodium alginate and whey protein concentrate; then concluded that the type of wall material affects both the rate of release of essential oil and the amount of essential oil that is encapsulated. Anyway, in all types of microcapsules made, some essential oil was placed on the surface of the wall apart from inside the core, but these amounts were different for each material which shows the importance of choosing the type of wall material. The method used in this research for essential oil release was diffusion based on the oily conditions of the environment. In fact, this group created a release medium containing 50% ethanol so that the essential oil on the wall and inside the core of the microcapsule is released into the environment based on the tendency to move toward lower concentrations of oil (Bajac et al., 2022).

## 8 ENCAPSULATION METHODS

Numerous strategies have been used to create and design microcapsules, like spray drying, freeze drying, electrospraying/electrospinning and inclusion complexation (Mohammadalinejhad & Kurek, 2021) that among them the spray drying method has been used the most in recent decades. The most important reason is the low cost of this strategy compared to other strategies but still has some advantages like easy to scale up, high speed, good storage stability and versatility (Furuta & Neoh, 2021; Ozkan et al., 2019). In a research conducted in 2022, the impact of hurdle technology using microencapsulated proteolytic enzymes and microencapsulated carvacrol, which is an effective ingredient in a variety of essential oils, was shown on the biofilm of *Pseudomonas aeruginosa* and *Enterococcus faecalis*. In this experiment, spray drying technique was used for microencapsulation. In short, imidazole‐acetate buffer was used to create buffer conditions and maintain the biological stability of the enzymes in such a way that after mixing the enzymes with pectin powder and maltodextrin, the resulting mixture was dissolved in the imidazole‐acetate buffer, adjusted to pH 7 and stored at room temperature. On the other hand, to make carvacrol emulsion, carvacrol and sodium caseinate were dissolved in water, then mixed with maltodextran powder and finally adjusted to pH 7. After homogenizing each of these solutions, they were spray‐dried separately and stored at 4°C. Now, two types of microcapsules have been made, one of which contains proteolytic enzymes such as pepsin and the other contains carvacrol. To treat these microcapsules with biofilm, first the microcapsule containing the enzyme was exposed to the biofilm for 1 h at 37°C and then the microcapsule containing carvacrol was applied for 1 to 5 min at 20°C. The results were very impressive, which showed that the population of *Pseudomonas aeruginosa* biofilm had reduced by 5 log CFU mL‐1 and the population of *Enterococcus faecalis* biofilm had reduced by 4 log CFU mL‐1 which has been much more efficient compared to the separate use of each of these treatments (Mechmechani, 2022).

In fact, use of microencapsulation in this hurdle technology causes increasing the stability of enzymes and essential oils, long‐term activity of these biocides, reducing the non‐specific connections of essential oils with the matrix, timely and concentrated release, and as a result, increasing the efficiency of biofilm removal (Khelissa et al., 2021). These advantages are very critical in the use of enzymes because these molecules remove the biofilm with their biological activity, so they must be able to stay in favorable conditions until reaching the biofilm. Particularly, microencapsulation protects the enzyme from the risk of denaturation, dilution and a decrease in the catalytic level (Kudryashov et al., 2020). Here are two examples of the increased effectiveness of enzymes and essential oils in their encapsulated form compared to their free form; by microencapsulating the enzyme combination of cellulase, pectin lyase and pronase, a significant reduction of *Pseudomonas fluorescens* occurred and 81% of the cells were detached from the biofilm at 7 h. This time is much longer in the free form of this enzyme compound, and a lower percentage of cells is isolated (Tan et al., 2020). It has also been reported that the encapsulated cinnamon oil has a significant effect on the methicillin‐resistant *Staphylococcus aureus* biofilm and its biocidal activity is much improved compared to the free use of this oil due to the increase in the stability of the essential oil and the increase in the effective treatment time (Wang, Zhao, et al., 2023).

## 9 CONCLUSION

Biofilm is a very resistant microbial population, where many people die every year due to the lack of effective removal by conventional sterilization methods, and also causes irreparable damage to various industries, specifically food industry. In this article, two environmental‐friendly sterilization methods including enzymes and essential oils were proposed, each of which can be used separately as a biocide, but the combination of these two causes a very effective cooperation to remove biofilm (Hurdle technology). Since both of the mentioned methods have high sensitivity to achieve high functional efficiency, they should not be applied through conventional methods, and it is necessary to have a protective and control system such as the microcapsule system. The use of the microencapsulation method in this hurdle technology causes the effective access and removal of cells by encapsulated essential oils after digestion of the extracellular polymer matrix in the biofilm by encapsulated enzymes. It should be noted that enzymes usually are not economical in the industries, but in cases where the effective and safe removal of biofilm is preferable to the economy of the method, this green technology can be used. Also, this hurdle technology is a very new method, as a result, not much research has been done on it and there are many questions in its use; but this technology can be a potential method in removing biofilms, especially in high sensitivity cases like cases related to food preservation and its safety control.

## AUTHOR CONTRIBUTIONS

**Ayda Ghahari:** Conceptualization (equal); data curation (equal); investigation (equal); methodology (equal); validation (equal); visualization (equal); writing – original draft (equal). **Kianoush Khosravi‐Darani:** Conceptualization (equal); formal analysis (equal); investigation (equal); methodology (equal); supervision (equal); validation (equal); writing – original draft (equal); writing – review and editing (equal).

## ACKNOWLEDGEMENTS

The authors acknowledge scientific support of NNFTRI & NIGEB.

## FUNDING INFORMATION

None of the authors have any found for this paper.

## CONFLICT OF INTEREST STATEMENT

The authors do not have any kind of interest.

## Data Availability

The data that support the findings of this study are available on request from the corresponding author.
